# Translating the observed differences in interleukin-6 levels between some antiretroviral regimens into potential long-term risk of serious non-AIDS events: A modeling study

**DOI:** 10.3389/fimmu.2022.976564

**Published:** 2022-11-08

**Authors:** Sergio Serrano-Villar, Calvin Cohen, Jason V. Baker, Maria João Janeiro, Filipa Aragão, Kathleen Melbourne, Jose Luis Gonzalez, Laura Lara, Connie Kim, Santiago Moreno

**Affiliations:** ^1^ Hospital Universitario Ramón y Cajal, Infectious Diseases, Instituto de Investigación Sanitaria Ramón y Cajal (IRYCIS), Madrid, Spain; ^2^ CIBERInfec, Instituto de Salud Carlos III, Madrid, Spain; ^3^ HIV Medical Affairs, Gilead Sciences Inc., Foster City, CA, United States; ^4^ Division of Infectious Diseases, Hennepin Healthcare Research Institute, Minneapolis, MN, United States; ^5^ Department of Medicine, University of Minnesota, Minneapolis, MN, United States; ^6^ Maple Health Group, New York, NY, United States; ^7^ Incremental Action Consulting Lda, Lisbon, Portugal; ^8^ NOVA National School of Public Health, Public Health Research Centre, Universidade NOVA de Lisboa, Lisbon, Portugal; ^9^ HIV Medical Affairs, Gilead Sciences Inc., Madrid, Spain; ^10^ Department of Medicine, Alcalá University, Madrid, Spain

**Keywords:** antiretroviral, HIV, inflammation, interleukin-6, Markov, three-drug regimen, two-drug regimen

## Abstract

**Introduction:**

Variable levels of systemic inflammation are observed in people with HIV (PWH), but the clinical significance of differences among antiretroviral therapy (ART) regimens on associated levels of inflammatory markers is unclear. Based on data from previous epidemiologic studies that defined the predicted change in risk of serious non-AIDS events (SNAEs)/death by changes in interleukin-6 (IL-6) and D-dimer, we modeled the effects of differences in these markers between specific ART regimens on the long-term risk of clinical outcomes.

**Methods:**

We used a Markov model to compare the risk of SNAEs/death with differences in IL-6 and D-dimer levels associated with remaining on specific three-drug regimens versus switching to specific two-drug ART regimens over 5 years of treatment. We used IL-6 and D-dimer data based on trajectories over time from the randomized TANGO and observational AIR studies. Age at model entry was set at 39 years. The primary endpoint was the number needed to treat for one additional SNAE/death.

**Results:**

Over 3 years, PWH on one of the three-drug regimens studied were predicted to spend 13% more time in the low IL-6 quartile and 11% less time in the high IL-6 quartile compared with those on one of the two-drug regimens. Over 3 years, the predicted mean number of SNAEs/deaths per 100 PWH was 6.58 for a three-drug regimen associated with lower IL-6 levels versus 6.90 for a two-drug regimen associated with higher IL-6 levels. The number needed to treat for one additional SNAE/death among PWH receiving a two-drug versus three-drug regimen for 3 years was 81. Approximately 7,500 participants would be required for a 5-year clinical study to evaluate the accuracy of the model.

**Conclusions:**

Our Markov model suggests that higher IL-6 levels associated with switching from specific three- to two- drug ART regimens may be associated with an increase in the risk of SNAEs/death. Clinical studies are warranted to confirm or refute these results.

## Introduction

People with HIV (PWH) typically have higher levels of immune activation and systemic inflammation than those without (1, 2). Such elevations are observed despite effective antiretroviral therapy (ART) and preserved CD4+ counts, and may persist for life. Chronic immune activation and inflammation are associated with increased risks of serious non-AIDS events (SNAEs) and adverse outcomes (1), with impacts on life expectancy and quality of life (3, 4).

In 2014, UNAIDS established the ‘90-90-90’ target, aiming to achieve ≥90% rates of diagnosis, ART and viral suppression among PWH (5). The addition of good health-related quality of life in PWH with viral load suppression was proposed as a ‘fourth 90’ in 2016 (6). In 2020, the targets were updated to include 95% diagnosed, 95% on treatment, and 95% viral suppression by 2025, with at least 90% of PWH linked to services they need for their overall health and well-being (7). Among PWH on continuous ART, the spectrum of morbidity and mortality commonly reflects non-AIDS comorbidities (8, 9), and despite 95% targets, long-term elevations of inflammatory markers may have a detrimental clinical impact. In an analysis of data from PWH on suppressive oral ART in the continuous ART arms (without IL-2) of three large randomized studies – Strategies for Management of Antiretroviral Therapy (SMART) (10, 11), Evaluation of Subcutaneous Proleukin in a Randomized International Trial (ESPRIT), and Subcutaneous Recombinant, Human Interleukin-2 in HIV-Infected Patients with Low CD4+ Counts under Active Antiretroviral Therapy (SILCAAT) (12) – elevations in the inflammatory markers interleukin-6 (IL-6) and D-dimer were associated with higher risks of SNAEs (cardiovascular, hepatic or renal events, or malignancy) and death (8).

Levels of IL-6 have been analyzed in many studies of ART. Elevated IL-6 levels have been linked with adverse outcomes in the general population and PWH (13, 14). Different inflammatory responses in PWH receiving treatment may be due to the choice of ART regimen; differences in some inflammatory markers have been observed in randomized studies with ART switches (15–17). While IL-6 levels are widely accepted as a robust predictor of adverse outcomes, the clinical significance of differences among ART regimens in their levels of IL-6 and other inflammatory markers is unclear.

Guidelines for the treatment of PWH currently recommend a variety of two- or three-drug ART regimens (18, 19). The choice of regimen is influenced by a variety of factors such as tolerability, short- and long-term safety, cost, patient convenience, genetic barriers to resistance, and baseline laboratory results. However, our understanding of which ART regimens produce the best overall outcomes among PWH, including long-term risk for SNAEs, is incomplete. Levels of immune activation and systemic inflammation may differ among regimens, which can contribute to long-term clinical outcomes. These could be overlooked in clinical trials with limited follow-up time that are powered to detect differences in viral load. The open-label, randomized, multicenter, phase 3 TANGO study compared continuing treatment with a three-drug, tenofovir alafenamide (TAF)-based regimen versus switching to a two-drug regimen (dolutegravir/lamivudine [DTG/3TC]) (20). The three-drug regimen was associated with significantly lower levels of IL-6 (P=0.006 and P=0.039 at Week 48 and 144, respectively) (20–22). The AIR study, a nested study in the Spanish AIDS Research Network involving a prospective, multicenter cohort of PWH, reported similar results. IL-6 decreased over time among recipients of a three-drug boosted protease inhibitor (PI)-based or integrase strand transfer inhibitor (INSTI)-based regimen. After an inflection point 3 years from virologic suppression, IL-6 levels in recipients of the three-drug regimens were lower than those among individuals who had switched to a two-drug regimen (DTG + 3TC, DTG + rilpivirine [RPV], or a boosted PI + 3TC; P=0.012 for the difference between IL-6 trajectories). The results were unchanged in a subanalysis that excluded the PI group. Significant differences in the trajectories of high-sensitivity C-reactive protein (hsCRP) and D-dimer were also observed (23).

In the current analysis, we used data on IL-6 and D-dimer changes in the TANGO and AIR studies to mathematically model the degree to which observed differences in inflammatory marker levels between a three-drug ART regimen associated with lower IL-6 levels and a two-drug ART regimen associated with higher IL-6 levels could affect clinical outcomes in virologically suppressed PWH.

## Materials and methods

### Markov model

We created a Markov model to compare continued treatment of virologically suppressed PWH with a three-drug ART regimen for which two data sources found was associated with lower IL-6 levels versus switching to specific two-drug ART regimens that were associated with higher IL-6 levels. The model is based on observed differences in IL-6 and D-dimer levels between the three- and two-drug ART regimens studied in the TANGO and AIR studies. As such, the terms ‘lower IL-6’ and ‘higher IL-6’ regimens are used to refer to the specific respective three- and two-drug ART regimens used in these two studies and in this model. Participants in the TANGO study were screened for eligibility between January 18, 2018 and May 18, 2018; and participants in the AIR study initiated follow-up between January 02, 2004 and December 28, 2018.

Markov modeling (24, 25) provides a simple framework for linking biomarkers to outcomes over an extended time horizon when it is sufficient to work with average values within health states (in this case IL-6 quartiles), and when differences within those health states are not of key interest. The Markov framework assumes that clinical history is not relevant to inform the future (predicting clinical outcomes), i.e., patients’ future risk of an event depends on their current rather than previous IL-6 quartile. In our model, for each year, an individual could experience: i) no events, ii) an SNAE/death, or iii) death.

Health states at baseline were defined based on the distribution of IL-6 levels observed in the AIR study (23) (median 1.26; standard deviation [SD] 2.27; Serrano-Villar, data on file). Given the asymmetric nature of IL-6 distributions, four health states were created, adopting the quartiles of a gamma distribution as boundaries (‘low’, ‘low–medium’, ‘medium–high’ and ‘high’). The model adopts 48-week long cycles to match the annual visits with IL-6 data in the TANGO study. The risk of an event is influenced by IL-6 levels, and these differed between three-drug and two-drug ART regimens (8).

Our model was based on IL-6 data, including trajectories over time, from the TANGO and AIR studies (20, 21, 23). IL-6 data from TANGO were available from baseline to Week 144 and were used for the first 3 years, with IL-6 data from Weeks 144 to 288 of the AIR study used for subsequent time points. Quartiles for baseline IL-6 levels were defined according to the distribution in the AIR study cohort. Given the different nature of data from the TANGO and AIR studies, transition probabilities were calculated differently for the first and subsequent 3 years. TANGO provided ratios in mean IL-6 values for different time points/visits. These ratios were used to project the IL-6 distribution from one time point to the subsequent, by defining updated cut-offs of IL-6 quartiles. The ratios between previous and subsequent distributions (i.e., the proportion of individuals in each quartile in subsequent time points), were adopted as transition probabilities. In the AIR study, the slope of a regression of IL-6 values over time was used to define transitions across health states. Here, the distribution of IL-6 values was updated every cycle based on a slope adjusted for 48 weeks, therefore redistributing patients among health states. In accordance with the TANGO study, no changes over time in D-dimer levels were assumed. Thus, the model results were based both on the change in IL-6, as well as the lack of change in D-dimer. The TANGO data were from 741 PWH (three-drug regimen, n=372; two-drug regimen, n=369). The AIR study included 148 PWH from a large cohort who met the inclusion criterion and who had at least three plasma samples after HIV RNA suppression that were available for analysis (three-drug regimen, n=90; two-drug regimen, n=58**)**. The three- and two-drug regimens included in the studies are shown in **Table 1**. In the TANGO study, the median age was 39 years for the three-drug regimen and 40 years for the two-drug regimen, and men accounted for 91% and 93% of the two cohorts, respectively (**Table 1**). In AIR, the mean ages were 37 and 40 years, and 87% and 86% of the patients were male, respectively. The age at entry in the model was set at 39 years, representing a weighted average from the main publications reporting IL-6 levels among PWH receiving different ART regimens (8, 26, 27).

**Table 1:** **Baseline demographics and clinical characteristics of the source data cohorts^a^** (8, 20, 23).

Demographic/clinical characteristic	Three-drug ART regimen(‘lower IL-6’)	Two-drug ART regimen(‘higher IL-6’)
**TANGO study**		
N	372	369
Median (range) age, years	39 (18–73)	40 (20–74)
Male, n (%)	339 (91)	344 (93)
Median (range) CD4+ cell count, cells/mm^3^	720 (119–1,810)	682 (133–1,904)
ART regimens, n		
TAF-based	372	–
DTG/3TC	–	369
**AIR study**		
N	90^b^	58^b^
Mean (SD) age, years	37 (9)	40 (11)
Male, n (%)	78 (87)	50 (86)
Median (IQR) CD4+ cell count, cells/mm^3^	300 (151–373)	259 (112–382)
ART regimens, n (%)		
PI-based		
ABC+3TC+bDRV	7 (7.8)	–
TDF+F+bDRV	30 (33.3)	–
ABC+3TC+LPVr	1 (1.1)	–
F+TDF+ATVr	2 (2.2)	–
F+TAF+DRVc	2 (2.2)	–
3TC+bATV	–	2 (3.5)
3TC+bDRV	–	13 (22.4)
INSTI-based		
ABC+3TC+DTG	24 (26.7)	–
ABC+3TC+RAL	7 (7.8)	–
F+TAF+EVGc	4 (4.4)	–
F+TDF+EVGc	4 (4.4)	–
F+TDF+DTG	2 (2.2)	–
F+TDF+RAL	7 (7.8)	–
DTG+3TC	–	7 (12.1)
DTG+RPV	–	36 (62.1)
	**ART regimen**	
**Analysis of SMART, ESPRIT, and SILCAAT studies**		
N	3,766	
Median age, years	42	
Female, %	21	
Median CD4+ cell count, cells/mm^3^	500	

3TC, lamivudine; ABC, abacavir; ATVr, atazanavir/ritonavir; bATV, boosted atazanavir; bDRV, boosted darunavir; DRVc, darunavir/cobicistat; DTG, dolutegravir; EVGc, elvitegravir/cobicistat; F, emtricitabine; IL-6, interleukin-6; LPVr, lopinavir/ritonavir; RAL, raltegravir; RPV, rilpivirine; SNAE, serious non-AIDS event; TAF, tenofovir alafenamide; TDF, tenofovir disoproxil fumarate.

^a^Model outputs were based on results from TANGO (N=741), the basis for the IL-6 distribution for the first three years, and results from the AIR study (N=148), which determined the distribution of patients across IL-6 health states during the following 3 years. While TANGO and AIR were adopted to distinguish between two- and three-drug ART regimens in terms of IL-6 progression, Grund et al., 2016 (8) (n=934, 949, 939, and 949 for IL-6 quartiles 4 [highest], 3, 2 and 1 [lowest], respectively) was adopted to inform the likelihood of SNAEs, conditional on IL-6 levels; ^b^Individuals selected with ≥3 stored plasma samples for analysis after HIV RNA suppression.

We derived the risk of SNAE/death from analyses of data from the INSIGHT trials network, which enabled the risk to be defined according to changes in IL-6 and D-dimer concentrations. The INSIGHT trials analyses included data from 3,766 virologically suppressed individuals from the control arms of the randomized SMART, ESPRIT, and SILCAAT studies (i.e., on continuous ART without IL-2), in which SNAEs and all-cause death were the primary endpoints (8). Demographic characteristics of PWH in the INSIGHT trials were similar to TANGO and AIR, with a high proportion of males (79% versus 91% and 93% in TANGO, and 87% and 86% in AIR in PWH on three- and two-drug ART regimens, respectively) and median age of 42 years (versus 39 and 40 years in TANGO and 37 and 40 years in AIR in PWH on three- and two-drug ART regimens, respectively) (**Table 1**) (8). The cumulative risk of events per IL-6 quartile was calculated assuming a lack of change over time in the D-dimer level, based on the results of the TANGO study (20, 21). This was a conservative assumption for longer-term changes in inflammation, considering the differences of higher D-dimer on two ART regimens as noted in AIR and a higher rate of events in the INSIGHT analysis associated with increases in D-dimer (23).

### Endpoints

The primary endpoint of the current analysis was the number needed to treat (NNT) for one additional SNAE/death at 144 and 240 weeks, based on the observed changes to IL-6. Predictions of time spent in each IL-6 quartile and numbers of SNAEs/deaths were considered as secondary endpoints, although these were required for calculation of the primary endpoint.

### Size of future clinical cohort

We estimated the sizes of clinical cohorts needed for a randomized clinical trial or an observational study to investigate the accuracy of the NNT results using the Markov model. A Poisson distribution was used to calculate sample sizes needed to detect differences in the number of SNAEs/deaths between regimens. A 2-sided test with a significance level (α) of 0.05 and a power of 0.8 was adopted (28).

### Ethics/participant consent

All clinical data used in this study were previously published, aggregated, and de-identified. Therefore, neither ethical approval nor patient consent was required or sought.

## Results

### Baseline characteristics

Baseline demographic and clinical characteristics of the source data cohorts are presented in **Table 1**. The median age and proportion of males were similar regardless of treatment regimen or study, but median CD4+ cell counts were higher in TANGO and INSIGHT studies than in the AIR study.

### IL-6 trajectories from the Markov model

**Figure 1:**
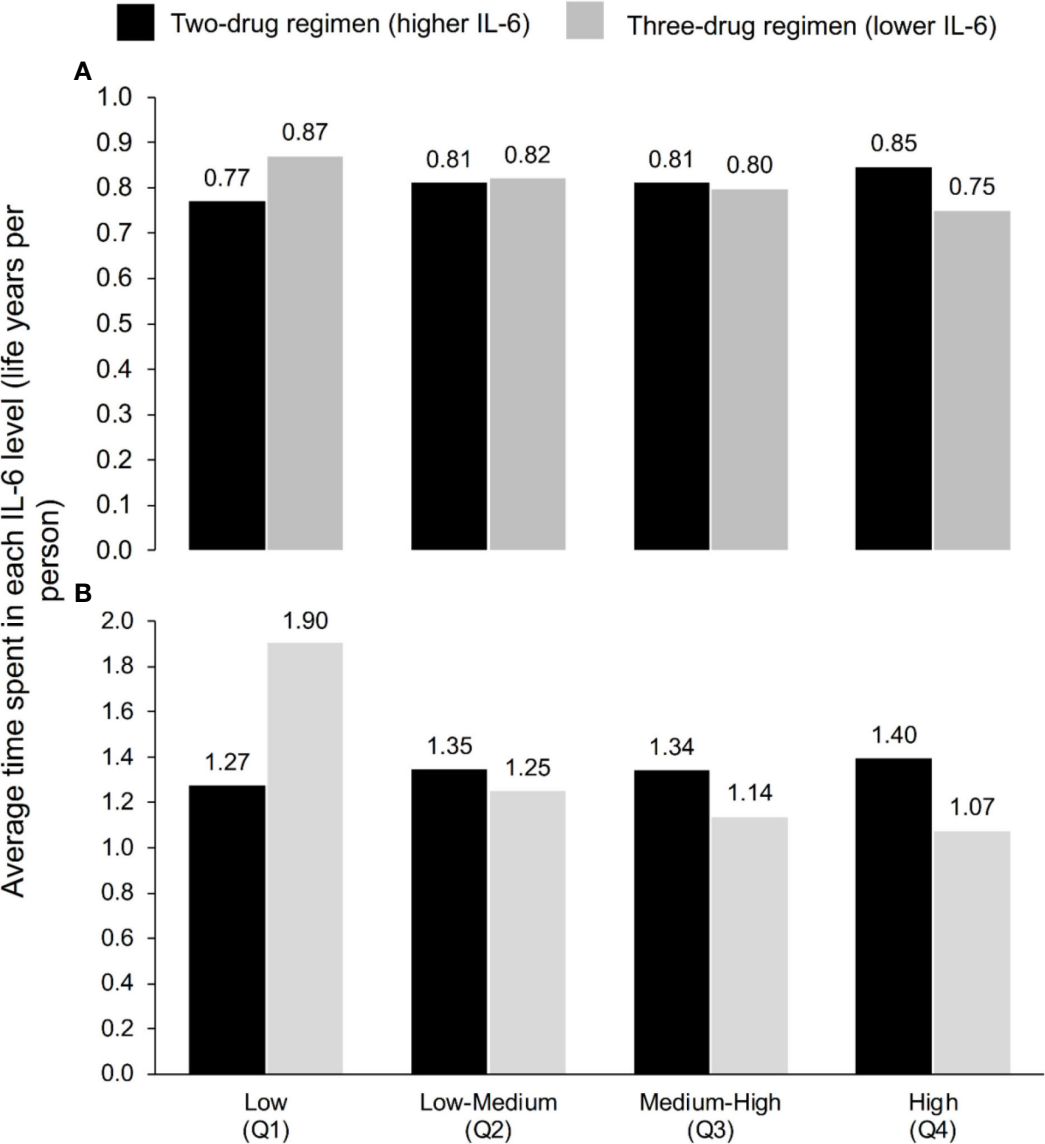
Average time spent in each IL-6 quartile according to ART regimen at **(A)** 3 years and **(B)** 5 years, according to the model, which used the IL-6 trajectories seen in TANGO (n=741, up to Week 144) and AIR (n=148, Weeks 144–240).

Within a time horizon of 3 years, PWH maintained on the three-drug ART regimens that were associated with lower IL6 levels (‘lower IL-6’ regimen) were predicted to spend 13% more time in the low IL-6 quartile and 11% less time in the high IL-6 quartile than those switching to a twodrug ART regimen associated with higher IL-6 levels (‘higher IL-6’ regimen) (**Figure 1A**). Differences between the two regimens in time spent in the middle two IL-6 quartiles were small. We observed a similar pattern over 240 weeks, although differences between the two regimens in the middle two quartiles were more pronounced (**Figure 1B**).

### Model-predicted SNAE/death rates and NNT

Overall, 260 participants in the INSIGHT studies experienced SNAE/death (8). The most common SNAEs/deaths were non-AIDS related cancer (37% of participants), cardiovascular disease (32%; including myocardial infarction [17%], stroke [7%], and deaths due to cardiovascular disease [7%]), decompensated liver cirrhosis (7%), end-stage renal disease (3%), and death due to other causes (21%; including AIDS [5%] and violence, accident or suicide [3%] (8).

**Figure 2:**
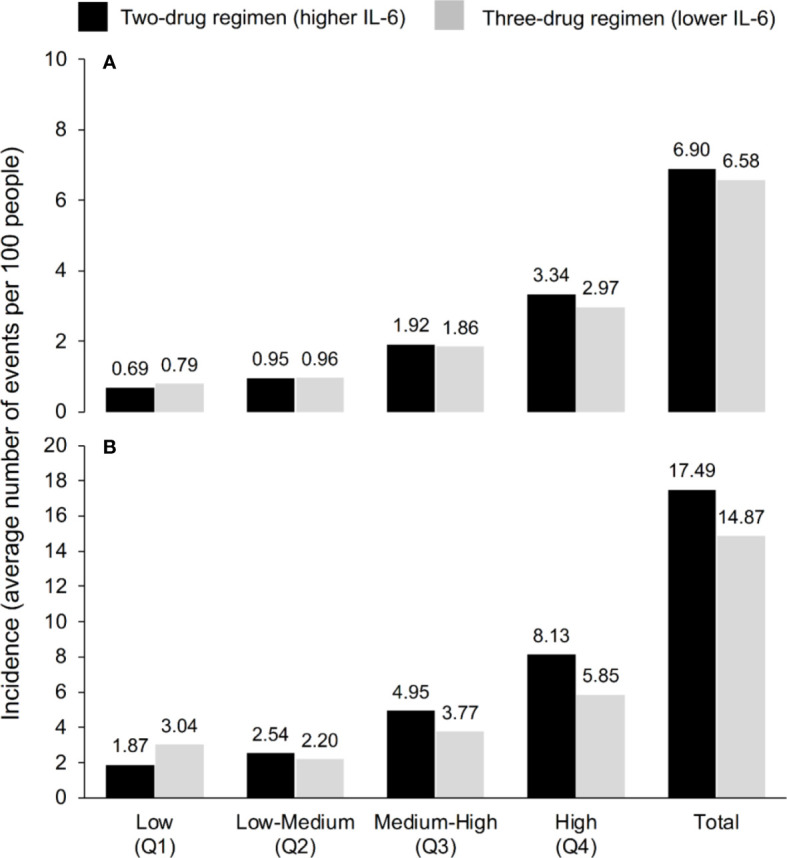
Incidence of serious non-AIDS events (cardiovascular, hepatic or renal event, or malignancy) or all-cause death according to IL-6 quartile and ART regimen over **(A)** 3 years and **(B)** 5 years, according to the model, which used the IL-6 trajectories seen in TANGO (n=741, up to Week 144) and AIR (n=148, Weeks 144–240).

Our model analysis of the 741 participants in the TANGO study showed that the predicted mean number of SNAEs/deaths per 100 PWH over 144 weeks was lower for continuous treatment with specific three-ART regimens versus switching to a two-ART regimen in three out of the four IL-6 quartiles (**Figure 2**). When all four IL-6 quartiles were combined, the predicted mean number of SNAEs/deaths per 100 PWH was 6.58 for continuous treatment with lower IL-6 compared with 6.90 with higher IL-6 (**Figure 2**). Results for the 5-year timeframe based on adding the 148 AIR study participants followed the same pattern, and the predicted mean numbers of SNAEs/deaths per 100 PWH were 14.87 and 17.49 for the three- and two-drug regimens, respectively. Based on these results, we calculated that for every 81 PWH treated for 5 years after switching to the two-drug regimen, there could be one additional SNAE/death as a consequence of higher IL-6, based on the association of IL-6 values and clinical outcomes from the INSIGHT studies. As shown in **Table 2**, the NNT decreased with increasing duration of treatment.

**Table 2:** Number needed to treat to observe one additional serious non-AIDS event (cardiovascular, hepatic or renal event, or malignancy) or all-cause death, with the two-drug ART regimen versus the three-drug regimen, by time on ART.

Time (years)	Number needed to treat
3	487
5	81
10	21

### Clinical trial size required to validate model-predicted results

To evaluate the accuracy of this model in a clinical setting, we first assumed that a two-arm study (either a randomized study or an observational cohort) could be performed with study participants receiving treatment with either a three- versus two-drug regimen (i.e., a regimen associated with a ‘lower versus ‘higher IL-6) in a 1:1 ratio. On this basis, 7,591 participants would be required for a study with a 5-year treatment period. Variations in the sample size according to the study duration are shown in **Table 3**. Different sample sizes would be required for unequal group sizes (i.e., allocation ratios other than 1:1). For example, a total of almost 8,500 patients would be needed for a 5-year study with a three- versus two-drug regimen ratio of 2:1.

**Table 3:** Estimated sample size required for a clinical study designed to support or refute the results of the Markov modeling.

Study length (years)	Sample size
3	214,041
5	7,591
10	656

## Discussion

Inflammatory markers such as IL-6, D-dimer and hsCRP are elevated in some PWH, even after virologic suppression has been achieved (2). In some studies, the levels of such markers appear to differ among ART classes and, depending on the specific regimens involved, they may be higher during treatment with some two-drug ART regimens after switching from some three-drug ART regimens (20–23). A range of factors may influence the level of systemic inflammation associated with an ART regimen. First, low-level HIV transcription in tissue reservoirs could trigger immune activation despite suppression of viral RNA levels in peripheral blood (29, 30). The extent of this phenomenon could be affected by the pharmacokinetics of the ART regimen (31). Second, the toxicity of certain ART regimens could increase the level of systemic inflammation to a greater extent than others, independent of the number of drugs being taken. Third, the ramifications of suboptimal adherence may lead to higher or more prolonged periods of low-level viremia in plasma, and this may differ according to the pharmacokinetic characteristics of different regimens. Suboptimal adherence has been associated with increased inflammation, even among individuals maintaining virologic suppression (32–34). Over the long term, high levels of inflammation have been associated with adverse clinical outcomes among PWH; this motivated the current study modeling potential clinical differences in suppressive regimens and based on observed differences in inflammatory markers. Higher levels of IL-6 and/or D-dimer have been reported to confer increased risk of SNAEs and death (8).

We constructed a Markov model to predict SNAE/death rates using data from the TANGO and AIR studies. These two studies were selected for their long follow-up durations and because they both provided data on IL-6 and D-dimer levels in people switching between ART regimens. Both studies reported higher IL-6 levels in people switching to the specific two-drug regimen(s) in use versus continuing treatment with several three-drug regimens. Our model suggested that switching from some three-drug ART regimens associated with lower IL-6 levels to some two-drug ART regimens associated with higher IL-6 levels may increase the risk of SNAEs and/or death in PWH. Specifically, one additional SNAE/death was predicted to occur for every 43 people treated for 240 weeks or for every 106 people treated for 144 weeks.

The results of our model are specific to the treatment regimens used in the TANGO trial and AIR study and cannot be extrapolated to other ART combinations. More recent two-drug regimens (e.g., long-acting injectable drugs with longer half-lives) may be associated with different levels of systemic inflammation. In other studies, switches from PI- to INSTI-based ART regimens were associated with differences in inflammatory markers, including decreases in not only IL-6 but also soluble CD14 (sCD14), intestinal fatty acid-binding protein, hsCRP, monocyte chemoattractant protein 1, osteoprotegerin, tumor necrosis factor α, and D-dimer (15–17), suggesting that differences in rates of SNAEs/deaths could also be observed among the regimens of these studies. Conversely, other clinical trial data found no differences in IL-6 levels over 48 weeks between patients switching from multiple three-drug regimens to DTG + 3TC versus those continuing three-drug regimens (35). While this suggests that no differences would be seen in the SNAE/death rates, the possibility remains that differences could emerge over a longer duration of follow-up or in subsets of the regimens used at entry.

IL-6 and other inflammatory markers are not routinely assessed in PWH, and the impact of inflammation on clinical outcomes is not clearly understood. International guidelines acknowledge the importance of inflammation in PWH but do not provide specific recommendations for the measurement, prevention, or treatment of inflammation (18, 19). This is partly because there is not enough evidence to show that interventions intended to change heightened inflammation levels affect clinical outcomes. This may also be in part due to the relatively small number of studies that examined differences in inflammatory markers with different regimens. Our study helps provide insight into the potential clinical consequences of increased inflammation associated with certain ART regimens, with guidance toward sample sizes of studies, including cohorts, that can be estimated to address this concern.

To contextualize the present results, we draw a comparison with statin therapy, an intervention proven to be beneficial when used at the population level. A review of data from 19 randomized trials showed that the NNT for statins to prevent composite cardiovascular outcomes over a treatment period of 1 to 6 years was 72 (36). In a Cochrane review, the adjusted 5-year NNT was 96 for prevention of death and 56 for prevention of a coronary heart disease event (either fatal or non-fatal) (37). Although the statin data are clearly defined by clinical outcomes in large numbers of patients, whereas the data in the present study are based on our model, the available evidence suggests that preferential use of ART regimens with reduced systemic inflammation could provide a magnitude of overall benefit consistent with other accepted medical interventions, such as statin therapy.

While the current study provides predictions based on the available data, additional data are warranted to refute or confirm the observed differences in inflammatory markers between regimens. In addition, clinical studies are necessary to refute or confirm the predicted effects on clinical outcomes of lower levels versus higher levels of inflammation while on ART. Our model indicates that randomized controlled or observational cohort trials would need to include large numbers of patients and a long treatment period (e.g., almost 3,000 patients for a two-arm, 5-year study). Considering the practical and financial challenges of conducting studies of this scale, a cohort-based approach appears more likely to provide additional clinical evidence to verify or refute these findings.

The principal strength of this study was the use of long-term results from large, parallel-group studies conducted in PWH. Thus, the risks of SNAEs/death were predicted from a robust clinical dataset. Another strength was the assumption of no changes in D-dimer levels (as seen in the shorter TANGO study); given the differences in D-dimer levels observed in the longer AIR study, and since both IL-6 and D-dimer have been found to independently contribute to the risk of severe non-AIDS events (38), this was a conservative approach for the longer-term consequences of changes in inflammation. In addition, as there were large differences in CD4+ counts between the source data cohorts, the results from the present model can be extrapolated to patients across a wide CD4+ count range.

Due to differences in study population and design, data from the TANGO and AIR studies from baseline to Week 144 were not combined. Other limitations include the theoretical nature of the model and consideration of only IL-6 (change) and D-dimer (lack of change) as inflammatory markers. We note that a significantly greater reduction in sCD14 was observed at two time points with two-drug versus three-drug ART regimens at 144 weeks in the TANGO study (20, 21, 39). The clinical relevance of this difference is uncertain, since an analysis of sCD14 in HIV infection showed that high sCD14 levels were associated with an increased risk of mortality but not with SNAEs, and only when results for both sCD14 and IL-6 changed in the same direction, in contrast to what was observed in TANGO (40). We are unaware of data that would have allowed us to model contradictory effects of these markers, so additional work would be needed to understand and incorporate that finding. However, there is evidence to show that IL-6 is the most robust inflammatory marker for predicting clinical events, irrespective of variations in other biomarkers (13). Epidemiologic data estimating their impact on incident SNAEs are less robust than the data for IL-6. An intrinsic limitation of the Markov model is that it does not account for the fact that inflammation over time could affect the disease outcomes. However, the START, SMART, ESPRIT and SILCAAT studies have demonstrated that a single measurement of inflammatory markers can predict long-term outcomes (up to 60 months and 9 years, respectively) (8, 26). We acknowledge that inflammation in PWH is multifactorial, and this study was focused only on the inflammatory effects of several specific ART regimens. The IL-6 data (and therefore the clinical outcomes) presented here may have limited applicability to female PWH due to the predominance of men in the TANGO and AIR studies, and may not be generalizable to older PWH, in whom the metabolism and elimination of ART may be slower than in younger PWH. Indeed, participant demographic differences may have affected levels of inflammatory markers, as may lifestyle factors; these were not considered in our model. Finally, our results should not be generalized to all two- and three-drug regimens; our results are only applicable to the two- and three-drug regimens associated with higher and lower IL-6, respectively, and in particular might not be generalizable to newer ART combinations.

In conclusion, our Markov model suggests that an increase in systemic inflammation associated with switching from a three-drug ART regimen associated with lower IL-6 levels to a two-drug ART regimen associated with higher IL-6 levels may increase the risk of SNAEs and/or death in PWH. Based on observed differences between regimens in IL-6 levels and no change to D-dimer as observed in the TANGO study, one additional SNAE/death was predicted to occur for every 43 PWH aged 30–50 years treated for 240 weeks with a regimen associated with higher IL-6 levels rather than maintaining a regimen associated with lower IL-6 levels. These predictions were based on the assumptions that an individual’s future risk of an event depends on their current rather than previous IL-6 quartile, and that variation in IL-6 levels in TANGO and AIR studies were due to differences in the ART regimens rather than other differences in, for example, ART adherence, comedication, classical risk factors or previous medical history. As TANGO was a large, randomized study, most of these factors are expected to be balanced between the arms. Nevertheless, it was an open-label study, and there may be other factors that have an impact on the observed findings on IL-6. Additional data sets are needed on these and other regimens to confirm or refute the inflammatory marker findings in TANGO and AIR. Finally, as this is a model, clinical studies would be needed to confirm our results. However, if verified, the selection of ART regimens associated with lower levels of systemic inflammation may become an important factor when optimizing long-term care of PWH.

## Data availability statement

The datasets presented in this article are not readily available because Gilead Sciences shares data upon request or as required by law or regulation with qualified external researchers based on submitted curriculum vitae and reflecting non-conflict of interest. The request proposal must also include a statistician. Approval of such requests is at Gilead Sciences’ discretion and is dependent on the nature of the request, the merit of the research proposed, the availability of the data, and the intended use of the data. Requests to access the datasets should be directed to Gilead Sciences, datarequest@gilead.com.

## Ethics statement

Ethical review and approval was not required for the study on human participants in accordance with the local legislation and institutional requirements. Written informed consent for participation was not required for this study in accordance with the national legislation and the institutional requirements.

## Author contributions

SS-V, CC, and SM were involved in the study design and data collection. All authors contributed to the article and approved the submitted version.

## Funding

This is a collaborative work, funded by Gilead Sciences, Inc (Foster City, CA, USA).

## Acknowledgments

The authors thank the investigators and participants who provided data for the original studies. Medical writing support, including the development of a draft outline and subsequent drafts in consultation with the authors, assembling tables and figures, collating author comments, copyediting, fact checking, and referencing was provided by Emma McConnell, PhD and Heather Davies, PhD CMPP at Aspire Scientific (Bollington, UK), and funded by Gilead Sciences, Inc (Foster City, CA, USA).

## Conflict of interest

SS-V received research grants from Gilead Sciences and MSD; non-financial support or fees from consulting or preparation of educational materials from Gilead Sciences, Janssen Cilag, MSD and ViiV Healthcare. CC, KM, JLG, LL and CK are employed by and own stocks/shares in Gilead Sciences. FA was employed by Maple Health Group. JVB received a research grant from Gilead for a study of remdesivir, 2020. SM has been involved in speaking activities and received grants for research from Gilead, Janssen Cilag, Merck, Sharp & Dohme and ViiV Healthcare.

The remaining author declares that the research was conducted in the absence of any commercial or financial relationships that could be construed as a potential conflict of interest.

The authors declare that this study received funding from Gilead Sciences. The funder had the following involvement with the study: the study design, collection, analysis, interpretation of data, the writing of this article and the decision to submit it for publication.

## Publisher’s note

All claims expressed in this article are solely those of the authors and do not necessarily represent those of their affiliated organizations, or those of the publisher, the editors and the reviewers. Any product that may be evaluated in this article, or claim that may be made by its manufacturer, is not guaranteed or endorsed by the publisher.
