# Corrigendum: Translating the observed differences in interleukin-6 levels between some antiretroviral regimens into potential long-term risk of serious non-AIDS events: A modeling study

**DOI:** 10.3389/fimmu.2023.1148980

**Published:** 2023-02-16

**Authors:** Sergio Serrano-Villar, Calvin Cohen, Jason V. Baker, Maria João Janeiro, Filipa Aragão, Kathleen Melbourne, Jose Luis Gonzalez, Laura Lara, Connie Kim, Santiago Moreno

**Affiliations:** ^1^ Hospital Universitario Ramón y Cajal, Infectious Diseases, Instituto de Investigación Sanitaria Ramón y Cajal (IRYCIS), Madrid, Spain; ^2^ CIBERInfec, Instituto de Salud Carlos III, Madrid, Spain; ^3^ HIV Medical Affairs, Gilead Sciences Inc., Foster City, CA, United States; ^4^ Division of Infectious Diseases, Hennepin Healthcare Research Institute, Minneapolis, MN, United States; ^5^ Department of Medicine, University of Minnesota, Minneapolis, MN, United States; ^6^ Maple Health Group, New York, NY, United States; ^7^ Incremental Action Consulting Lda, Lisbon, Portugal; ^8^ NOVA National School of Public Health, Public Health Research Centre, Universidade NOVA de Lisboa, Lisbon, Portugal; ^9^ HIV Medical Affairs, Gilead Sciences Inc., Madrid, Spain; ^10^ Department of Medicine, Alcalá University, Madrid, Spain

**Keywords:** antiretroviral, HIV, inflammation, interleukin-6, Markov, three-drug regimen, two-drug regimen

In the published article, there was an error in **Tables 2** and **3** and **Figures 1** and **2** as published. A formula correction was implemented in the.xlsm file containing the Markov model, leading to changes to the previously reported results. The corrected **Tables 2** and **3** and **Figures 1** and **2** and their captions appear below.

**Figure 1:**
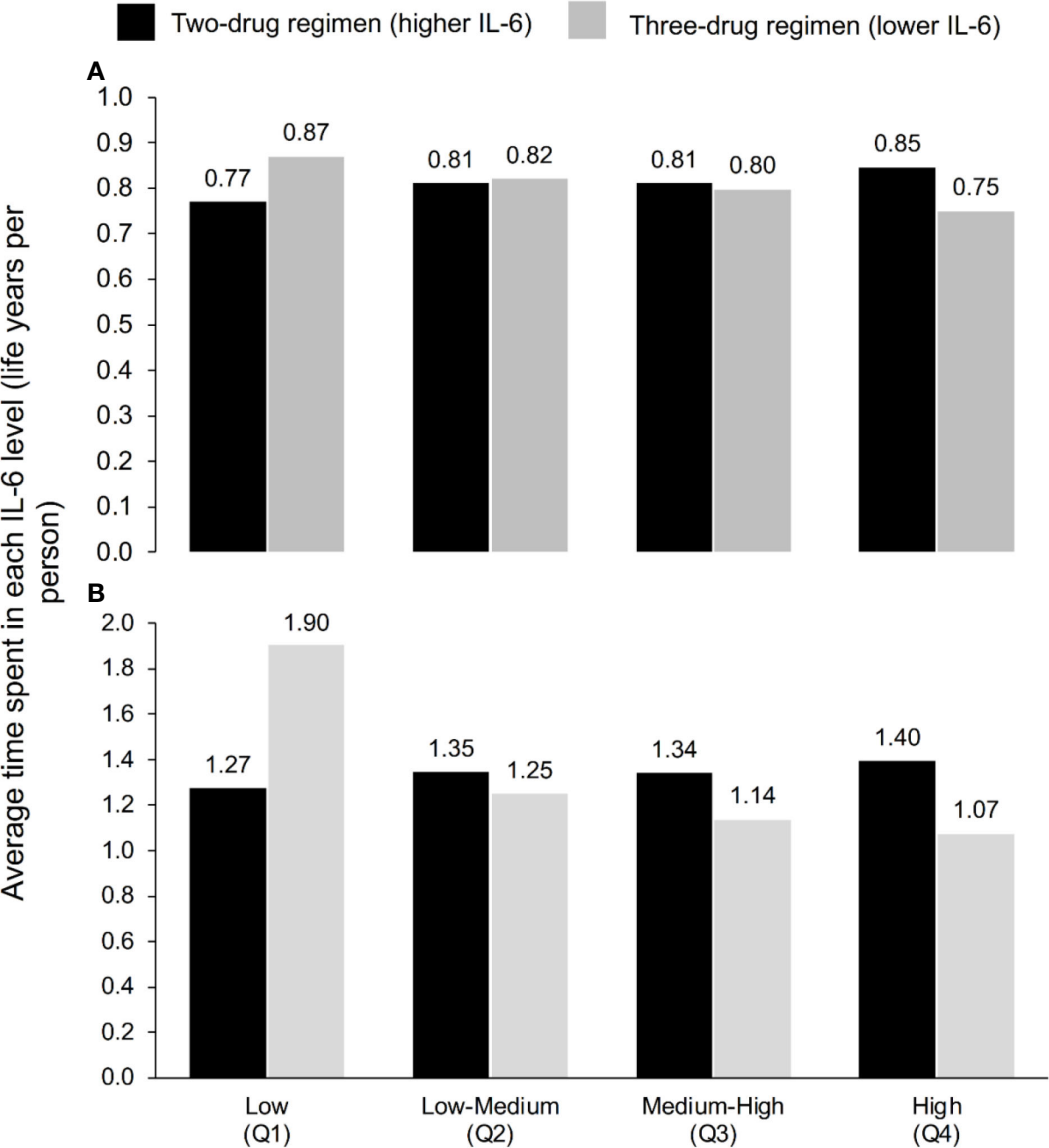
Average time spent in each IL-6 quartile according to ART regimen at **(A)** 3 years and **(B)** 5 years, according to the model, which used the IL-6 trajectories seen in TANGO (n=741, up to Week 144) and AIR (n=148, Weeks 144–240).

**Figure 2:**
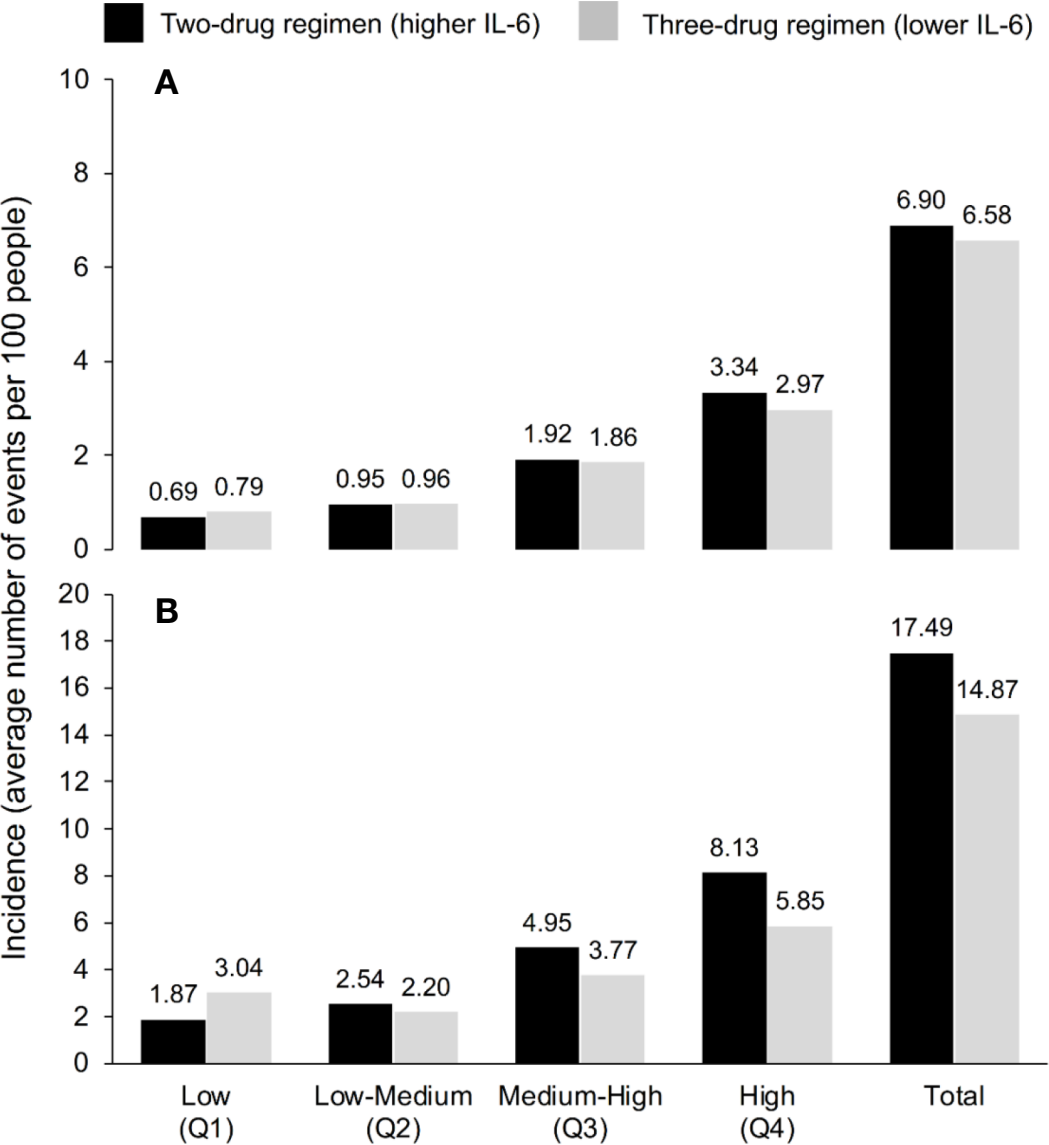
Incidence of serious non-AIDS events (cardiovascular, hepatic or renal event, or malignancy) or all-cause death according to IL-6 quartile and ART regimen over **(A)** 3 years and **(B)** 5 years, according to the model, which used the IL-6 trajectories seen in TANGO (n=741, up to Week 144) and AIR (n=148, Weeks 144–240).

**Table 2:** Number needed to treat to observe one additional serious non-AIDS event (cardiovascular, hepatic or renal event, or malignancy) or all-cause death, with the two-drug ART regimen versus the three-drug regimen, by time on ART.

Time (years)	Number needed to treat
3	487
5	81
10	21

**Table 3:** Estimated sample size required for a clinical study designed to support or refute the results of the Markov modeling.

Study length (years)	Sample size
3	214,041
5	7,591
10	656

Corrections have also been made to the **Abstract**, **Results**, Paragraph 1. This paragraph previously stated:

“Over 144 weeks, PWH on one of the three-drug regimens studied were predicted to spend 22% more time in the low IL-6 quartile and 13% less time in the high IL-6 quartile compared with those on one of the two-drug regimens. Over 144 weeks, the predicted mean number of SNAEs/deaths per 100 PWH was 5.6 for a three-drug regimen associated with lower IL-6 levels versus 6.8 for a two-drug regimen associated with higher IL-6 levels. The number needed to treat for one additional SNAE/death among PWH receiving a two-drug versus three-drug regimen for 240 weeks was 43. Approximately 2,900 participants would be required for a 240-week clinical study to evaluate the accuracy of the model.” The corrected paragraph appears below:

“Over 3 years, PWH on one of the three-drug regimens studied were predicted to spend 13% more time in the low IL-6 quartile and 11% less time in the high IL-6 quartile compared with those on one of the two-drug regimens. Over 3 years, the predicted mean number of SNAEs/deaths per 100 PWH was 6.58 for a three-drug regimen associated with lower IL-6 levels versus 6.90 for a two-drug regimen associated with higher IL-6 levels. The number needed to treat for one additional SNAE/death among PWH receiving a two-drug versus three-drug regimen for 3 years was 81. Approximately 7,500 participants would be required for a 5-year clinical study to evaluate the accuracy of the model.”

A correction has been made to the **Results**, **IL-6 trajectories from the Markov model**, Paragraph 1. This sentence previously stated: “Within a time horizon of 144 weeks, PWH maintained on the three-drug ART regimens that were associated with lower IL6 levels (‘lower IL-6’ regimen) were predicted to spend 22% more time in the low IL-6 quartile and 13% less time in the high IL-6 quartile than those switching to a two-drug ART regimen associated with higher IL-6 levels (‘higher IL-6’ regimen) (Figure 1A).” The corrected sentence appears below:

“Within a time horizon of 3 years, PWH maintained on the three-drug ART regimens that were associated with lower IL6 levels (‘lower IL-6’ regimen) were predicted to spend 13% more time in the low IL-6 quartile and 11% less time in the high IL-6 quartile than those switching to a two-drug ART regimen associated with higher IL-6 levels (‘higher IL-6’ regimen) (Figure 1A).”

Corrections have been made to the **Results**, **Model-predicted SNAE/death rates and NNT**, Paragraph 2.

(1) This sentence previously stated:

“When all four IL-6 quartiles were combined, the predicted mean number of SNAEs/deaths per 100 PWH was 5.6 for continuous treatment with lower IL-6 compared with 6.8 with higher IL-6 (Figure 2).” The corrected sentence appears below:

“When all four IL-6 quartiles were combined, the predicted mean number of SNAEs/deaths per 100 PWH was 6.58 for continuous treatment with lower IL-6 compared with 6.90 with higher IL-6 (Figure 2).”

(2) This sentence previously stated: “Results for the 240-week timeframe based on adding the 148 AIR study participants followed the same pattern, and the predicted mean numbers of SNAEs/deaths per 100 PWH were 11.9 and 15.8 for the three- and two-drug regimens, respectively.” The corrected sentence appears below:

“Results for the 5-year timeframe based on adding the 148 AIR study participants followed the same pattern, and the predicted mean numbers of SNAEs/deaths per 100 PWH were 14.87 and 17.49 for the three- and two-drug regimens, respectively.”

(3) This sentence previously stated: “Based on these results, we calculated that for every 43 PWH treated for 240 weeks after switching to the two-drug regimen, there could be one additional SNAE/death as a consequence of higher IL-6, based on the association of IL-6 values and clinical outcomes from the INSIGHT studies.” The corrected sentence appears below:

“Based on these results, we calculated that for every 81 PWH treated for 5 years after switching to the two-drug regimen, there could be one additional SNAE/death as a consequence of higher IL-6, based on the association of IL-6 values and clinical outcomes from the INSIGHT studies.”

Corrections have been made to the **Results**, **Clinical trial size required to validate model-predicted results**, Paragraph 1. (1) This sentence previously stated: “On this basis, 2,906 participants would be required for a study with a 240-week treatment period. Variations in the sample size according to the study duration are shown in Table 3.” The corrected sentence appears below:“On this basis, 7,591 participants would be required for a study with a 5-year treatment period. Variations in the sample size according to the study duration are shown in Table 3." (2) This sentence previously stated: “For example, a total of almost 10,000 patients would be needed for a 240-week study with a three- versus two-drug regimen ratio of 2:1.”The corrected sentence appears below:“For example, a total of almost 8,500 patients would be needed for a 5-year study with a three- versus two-drug regimen ratio of 2:1.”The authors apologize for these errors and state that they do not change the scientific conclusions of the article in any way. The original article has been updated.

